# A Descriptive Study of Open Fractures Contaminated by Seawater: Infection, Pathogens, and Antibiotic Resistance

**DOI:** 10.1155/2017/2796054

**Published:** 2017-02-20

**Authors:** Hongyi Zhu, Xingwei Li, Xianyou Zheng

**Affiliations:** Department of Orthopaedic Surgery, Shanghai Jiaotong University Affiliated Sixth People's Hospital, Shanghai 200233, China

## Abstract

*Aims*. In this work, the main objectives were to investigate the clinical characteristics and bacterial spectrum present in open fractures contaminated by seawater.* Methods*. We conducted a retrospective cohort study and included all patients with open fractures from 1st January, 2012, to 31st December, 2015, in our hospital. Patients were grouped based on the presence of seawater contamination in wounds. We compared the infection rate, bacterial spectrum, and antibiotic resistance between the two groups.* Results*. We totally included 1337 cases of open fracture. Wounds from 107 cases (8.0%) were contaminated by seawater. The wound infection rate of seawater-contaminated group was significantly higher in patients with Gustilo-Anderson Type II and Type III open fractures. The bacterial spectrum from seawater-contaminated wounds was remarkably different from that of the remaining. Antibiotic sensitivity tests revealed that more than 90% of infecting pathogens in seawater-contaminated wounds were sensitive to levofloxacin and ciprofloxacin.* Conclusion*. Cephalosporin in combination with quinolone was recommended in the early-stage management of open fractures contaminated by seawater.

## 1. Introduction

The management of wounds associated with open fractures has been a continuing source of controversy within the orthopedic society [1, 2]. Damage in soft tissues and local perfusion represents major issues in treatment of open fractures. For the assessment of open fractures, the Gustilo-Anderson classification system was widely used [3]. Depending on severity of the lesion, treatment might include debridement and internal and external fixation in the acute management [4, 5]. The following bacterial infection of bony and soft tissues is a major challenge which might further lead to sepsis, acute or chronic osteomyelitis, delayed union, nonunion, or even loss of the extremity. The extent of infection and the involved bacterial species depend on host, injury mechanism, the severity of injury, and posttrauma management [6, 7]. Antibiotics play an important role in the treatment of infection related to open fractures. In addition, a prospective, randomized trial in 1104 patients with open fractures demonstrated a significant reduction in infection rate with prophylactic administration of antibiotics [8].

Wound bacterial cultures before surgical debridement were routinely performed prior to the 1980s [9, 10]. However, the value of this practice has been questioned in recent years. Multiple studies have revealed that the predebridement wound cultures had minimal values in predicting the postdebridement wound infection since the infecting pathogens were commonly hospital-acquired [11–16]. As a result, the choice of antibiotics in early-stage management was mainly based on published evidences and cephalosporin was most widely adopted and recommended [7, 17, 18].

## 2. Patients and Methods

This study was approved by our institutional ethics board. The inclusion criteria for this study were an open fracture of any type involving any extremity, for which the entire fracture management was under the care of our orthopedic department. Patients with additional injuries on other organs were excluded. Finally, we identified a total of 1337 cases with complete data set presenting to our hospital with open fractures from 1st January, 2012, to 31st December, 2015. All clinical information was collected from medical records. Seawater contamination was defined as the condition for which the wounds were contacted with seawater directly and indirectly. Seawater contamination was presented in 107 cases (8.0%).

All patients were initially evaluated in the emergency department and Gustilo-Anderson classification system was used [3]. After initial evaluation, all open wounds were irrigated with saline solution and dressed with sterile gauze. The limb was then immobilized in an appropriate fashion to facilitate further patient management. The patients were routinely given tetanus prophylaxis. All patients received intravenous cephalosporin antibiotics immediately in the emergency department. The patients were taken to the operating room for further wound irrigation and debridement and stabilization of the fracture. The decision to leave the wound open or closed was made by treating surgeons and based on the degree of soft-tissue damage. All treating surgeons were specialists in traumatology. Open wounds were sealed with a negative-pressure closure device. The initial culture was not taken due to the minimal values of this practice. The antibiotic sensitivity tests at least included imipenem, cefazolin, cefotetan, ceftriaxone, gentamycin, amikacin, ampicillin, levofloxacin, ciprofloxacin, and piperacillin/tazobactam. The patient subsequently returned to the operating room for additional irrigation and debridement if necessary. The samples for bacterial culture were obtained before each time of additional irrigation and debridement. A negative-pressure closure dressing was also routinely applied after irrigation and debridement. The interval of two surgical debridement instances varied from three to seven days. Wound closure would be attempted if the latest culture obtained was negative. Deep infection was defined as infection of deep tissues and systemic infection and abscess in other organs and osteomyelitis.

The significance of differences between groups in each variable was assessed using Pearson's chi-squared test or Student's *t*-test. All data were presented as mean ± standard deviation. The alpha level for significance was set at 0.05.

## 3. Results

One thousand three hundred and thirty-seven open fractures were identified during the study period. Ninety-seven patients had more than one open fracture, and each fracture was evaluated individually. The mean age was 39.1 ± 13.3 years (from sixteen to seventy-three years). There were 967 cases of males (72.3%) and 370 cases of females (27.7%). According to Gustilo-Anderson classification, there were 456 (34.1%) and 330 (24.7%) cases classified as Type I and Type II, respectively. Five hundred and fifty-one cases (41.2%) were Type III open fractures. Lower limb (55.2%) and upper limb (41.4%) were the most commonly injured locations. We divided all cases into two groups based on the presence of seawater contamination. The demographic and clinical features of the two groups were summarized in Table 1. The overall wound infection rate of seawater-contaminated wounds was significantly higher than that of wounds without seawater contamination (Table 2). In subgroups with different Gustilo-Anderson types, the wound infection rates of seawater-contaminated Type II, IIIA, IIIB, and IIIC fractures were remarkably increased compared with control groups. The deep infection was rare for Type I and Type II. Hence, we compared the deep infection rate of Type III fractures between two groups and the rate of deep infection was significantly higher in seawater contamination group (15.2% versus 5.0%, *P* = 0.03). Interestingly, the infected wounds in seawater contamination group required more times of surgical debridement before attempting wound closure (3.3 ± 1.5 versus 2.4 ± 1.1, *P* < 0.001).

In this study, a total of 251 cases required additional surgical debridement and samples for bacterial cultures were collected to detect the infecting pathogens and their sensitivities to antibiotics. The nosocomial pathogens including* Pseudomonas aeruginosa*,* Acinetobacter baumannii*,* Enterobacter,* and methicillin-resistant* Staphylococcus aureus* (MRSA) were predominant in infection of wounds without seawater contamination (Table 3). In contrast, there were no predominant pathogens and nosocomial pathogens were not commonly encountered in seawater-contaminated wounds. The sensitivity tests showed that imipenem, levofloxacin, and ciprofloxacin were the most effective antibiotics in treatment of infection in seawater-contaminated wounds (Table 4).

## 4. Discussion

The management of open fractures is challenging. Infection is a major complication of open fractures. In this study, we attempted to investigate the characteristics of infection and associated pathogens in the seawater-contaminated open fracture. The wound infection rates of seawater-contaminated Type II, IIIA, IIIB, and IIIC fractures were remarkably increased compared with control groups. It appeared that the fractures most impacted by seawater contamination were the severe Type III injuries. The impressively high rates of wound infection for this group in our study may have been related to the different pathogen spectrum. Consistently, the deep infection rate was significantly higher in seawater contamination group. These findings suggested the infection in seawater-contaminated wounds was refractory and challenging for management.

The choice of prophylactic antibiotics for open fractures was important for the management of open fracture. However, the previous studies showed controversial results of optimal therapy. One major reason was that hospital-acquired pathogens were increasingly present in the infection of open fractures and the antibiotic resistance of nosocomial pathogens varied among different institutes. Consistent with previous studies, hospital-acquired pathogens were also predominantly responsible for the infection of wounds without seawater contamination in our study. As a result, no antibiotic except imipenem showed dominant coverage to these pathogens. In contrast, there were no predominant pathogens and nosocomial pathogens were not commonly encountered in seawater-contaminated wounds. The sensitivity tests showed that imipenem, levofloxacin, and ciprofloxacin were the most effective antibiotics in treatment of infection in seawater-contaminated wounds. Since imipenem was appropriate for antibiotic prophylaxis and regular treatment for infection, we recommended prophylactic administration of cephalosporin and quinolone in the early-stage management of open fractures contaminated by seawater. Notably, it is still unknown whether prophylactic administration of this combination could decrease the infection rate in seawater-contaminated open fractures compared with cephalosporin alone. Further study is required to address this question.

More times of surgical debridement were required once infection was presented in seawater-contaminated wounds. The reason remained unclear. Although the debridement was thorough and meticulous, the adhesion of remaining pathogens could not be entirely avoided. One possibility was that the adhesion of the infecting pathogens was stronger. In addition, based on our experience, the host tissue destruction was commonly more severe in seawater-contaminated wounds. The necrotic tissues clearly posed an obstacle to the eradication of infecting pathogens by host immune system.

## Figures and Tables

**Table 1:** Demographic and clinical characteristics.

	Seawater contamination	No seawater contamination	*P* value
	(*n* = 107)	(*n* = 1230)	
Age	40.6 ± 11.4	39.0 ± 13.5	0.17
Gender			
Male	77 (72)	890 (72)	0.93
Female	30 (28)	340 (28)	
Gustilo-Anderson classification			
I	40 (37)	416 (34)	0.36
II	21 (20)	309 (25)	
IIIA	11 (10)	141 (11)	
IIIB	14 (13)	193 (16)	
IIIC	21 (20)	171 (14)	
Location of open fracture			
Upper limb	46 (43)	508 (41)	0.13
Lower limb	61 (57)	677 (55)	
Another location	0 (0)	45 (4)	

Figures are numbers (percentage).

**Table 2:** Wound infection rate of open fractures with different Gustilo-Anderson types grouped by seawater contamination.

Gustilo-Anderson classification	Seawater contamination	Wound infection		*P* value
		No	Yes	
I	No	394 (95)	22 (5)	0.38
	Yes	39 (98)	1 (2)	
II	No	274 (89)	35 (11)	0.03
	Yes	15 (71)	6 (29)	
IIIA	No	92 (65)	49 (35)	0.02
	Yes	3 (27)	8 (73)	
IIIB	No	118 (61)	75 (39)	0.02
	Yes	4 (29)	10 (71)	
IIIC	No	95 (56)	76 (44)	<0.001
	Yes	4 (19)	17 (81)	
Overall	No	973 (79)	257 (21)	<0.001
	Yes	65 (61)	42 (39)	

**Table 3:** Infecting pathogens in wounds grouped by seawater contamination.

No seawater contamination		Seawater contamination	
(*n* = 210)		(*n* = 41)	
*Pseudomonas aeruginosa*	46	*Vibrio*	4
*Enterobacter*	34	*Providencia*	3
*Staphylococcus aureus*	28	*Mycobacterium*	3
*Escherichia coli*	25	*Pseudomonas aeruginosa*	2
MRSA	20	*Bacaeroides*	2
*Acinetobacter baumannii*	16	*Morganella*	2
*Enterococcus*	14	*Staphylococcus aureus*	2
*Proteus mirabilis*	11	*Proteus mirabilis*	2
Others	16	Others	21

Total	210	Total	41

**Table 4:** Antibiotic sensitivity in wounds grouped by seawater contamination.

	Sensitive rate	
	No seawater contamination	Seawater contamination
	(*n* = 210)	(*n* = 41)
Imipenem	206 (98)	41 (100)
Levofloxacin	121 (58)	39 (95)
Ciprofloxacin	119 (57)	38 (93)
Cefazolin	104 (50)	22 (54)
Cefotetan	122 (58)	25 (61)
Ceftriaxone	121 (58)	27 (66)
Gentamycin	168 (80)	30 (73)
Amikacin	170 (81)	29 (71)
Ampicillin	88 (42)	24 (59)
Piperacillin/tazobactam	106 (50)	27 (66)

Figures are numbers (percentage).
